# Mr. Leonard Hill on Dr. Lauder Brunton

**Published:** 1898-03-26

**Authors:** 


### MR. LEONARD HILL ON DR. LAUDER BRUNTON.

Mr. Leonard Hill has not been long in answering Dr. Lauder Brunton, and it is impossible not to see that so far he has the best of it. Mr. Hill first suggests original bias in the Hyderabad Commission: "The first Commission 'was applied for by Surgeon-Major Lawrie because, having always believed in the truth of Syme's teaching that chloroform can be used judiciously so as to do good without the risk of evil, he desired to show by experiment upon dogs that in death from chloroform the respiration always stops before the heart.' This Commission determined, among other things, that 'with the cessation of respiration the further accumulation of the drug in the blood necessarily ceases, and the heart rapidly or gradually stops beating as a direct result of the stoppage of respiration.' 'This point having been decided the second Commission was applied for because it was felt that Symes' principles, which both experience and experiment had shown to be practically sound, must be founded upon a firm physiological basis.'" Then Mr. Hill rubs in the obvious weakness of Dr. Brunton's case, which arises from the admitted shortness of the time devoted to the investigation—namely, 47 days—and in regard to this asks, "Why, if 'with the exception of time nothing was left to be desired,' was not the three months' ticket extended?" as had been provided for in the arrangements. Mr. Hill entirely disclaims any intention of personally attacking Dr. Brunton in anything that he has said about the Hyderabad Commission, maintaining that he could not be expected to imagine that the whole Commission was personated by Dr. Brunton, and he insists that, considering the gravity of the subject and the "Oriental pomp with which the work of the second Commission was thrust upon the scientific world," he was justified in attacking with vigour a doctrine which he believed to be erroneous and dangerous. His attack, however, was upon the Commission, not upon Dr. Lauder Brunton, and if his language was "vigorous," he shows that the language of the Commission, and of Dr. Brunton in particular, in regard to other investigators, was quite open to the same charge. Mr. Hill then enters upon his justification of the charge of carelessness which he brought against the Commission, showing that in several instances they failed to adopt precautions in the conduct of experiments which are now recognised to be essential in arriving at correct results. Finally, he says: "I am in entire sympathy with Dr. Brunton when he writes—'It would have been advisable to repeat several experiments if we had time, and it might have been advantageous on several occasions to use other apparatus if it had been available,'" all of which is rather neat!

